# Identifying Robust Microbiota Signatures and Interpretable Rules to Distinguish Cancer Subtypes

**DOI:** 10.3389/fmolb.2020.604794

**Published:** 2020-11-04

**Authors:** Lei Chen, Zhandong Li, Tao Zeng, Yu-Hang Zhang, Dejing Liu, Hao Li, Tao Huang, Yu-Dong Cai

**Affiliations:** ^1^School of Life Sciences, Shanghai University, Shanghai, China; ^2^College of Information Engineering, Shanghai Maritime University, Shanghai, China; ^3^College of Food Engineering, Jilin Engineering Normal University, Changchun, China; ^4^Zhangjiang Laboratory, Institute of Brain-Intelligence Technology, Shanghai, China; ^5^Channing Division of Network Medicine, Brigham and Women’s Hospital, Harvard Medical School, Boston, MA, United States; ^6^Key Laboratory of Tissue Microenvironment and Tumor, Shanghai Institute of Nutrition and Health, Chinese Academy of Sciences, Shanghai, China

**Keywords:** cancer type, microbiota, machine learning algorithm, decision tree, rules

## Abstract

Cancer can be generally defined as a cluster of systematic diseases triggered by abnormal cell proliferation and growth. With the development of biological sciences and biotechnologies, the etiology of cancer is partially revealed, including some of the most substantial pathogenic factors [either endogenous (genetics) or exogenous (environmental)]. However, some remaining factors that contribute to the tumorigenesis but have not been analyzed and discussed in detail remain. For instance, some typical correlations between microorganisms and tumorigenesis have been reported already, but previous studies are just sporadic studies on single microorganism–cancer subtype pairs and do not explain and validate the specific contribution of microbiome on tumorigenesis. On the basis of the systematic microbiome analyses of blood and cancer-associated tissues in cancer patients/controls in public domain, we performed interpretable analyses. We identified several core regulatory microorganisms that contribute to the classification of multiple tumor subtypes and established quantitative predictive models for interpretable prediction by using multiple machine learning methods. We also compared the optimal features (microorganisms) and rules identified from microbiome profiles processed using the Kraken and the SHOGUN. Collectively, our study identified new microbiome signatures and their interpretable classification rules for cancer discrimination and carried out reliable methodological comparison for robust cancer microbiome analyses, thereby promoting the development of tumor etiology at the microbiome level.

## Introduction

Cancer, as one of the most threatening diseases all over the world, can be generally defined as a cluster of systematic diseases triggered by abnormal cell proliferation and growth (McGuire, 2016; Vanhoutte et al., 2016). According to the World Health Organization (Vanhoutte et al., 2016; Shams-White et al., 2019), cancer is the second leading cause of death compared with other diseases and causes nearly 10 million deaths and about 20 million newly reported cases worldwide in 2018. China has comparable cancer morbidity and a relatively quite high mortality with the world average, and about 4 million new cancer cases and 3 million cancer-associated deaths are reported in 2018 from China (Feng et al., 2019; Shams-White et al., 2019), indicating that cancer is one of the most threatening diseases in China.

With the development of biological sciences and the progress of biotechnologies, the etiology of cancer is partially revealed, including some of the most significant pathogenic factors [either endogenous (genetics) or exogenous (environmental)]. In previous studies, the endogenous [like genes *EGFR* (Wang et al., 2019), *TP53* (Salk et al., 2019), and *RAS* (Lanfredini et al., 2019)] and the exogenous [like smoking (Croyle et al., 2019), alcoholism (Srivastava et al., 2019) and severe air pollutions (Guo et al., 2019)] factors are widely reported to participate in tumor-associated biological processes, some of which are reported to directly trigger the initiation of tumorigenesis. Current studies are systematic and thorough, but some remaining factors that contribute to the tumorigenesis but are not analyzed and discussed in detail remain.

The relationships between microorganisms and cancers have been reported for decades. For viruses, in the seventh decade of the 20th century, the infection of the hepatitis B virus (HBV) is correlated with the initiation and the progression of hepatocellular carcinoma after a long course of HBV infections (Feng et al., 2019). Two types of human papillomavirus, i.e., HPV-16 and HPV-18, are identified as the most important pathogens for cervical cancers (Shibata et al., 2019). Vaccines against HPV-16 and HPV-18 are developed and promoted among adolescent girls and adult women to prevent the high incidence of cervical cancers (Di Bonito et al., 2019; Shibata et al., 2019). Apart from the virus, some bacteria are functionally correlated with certain cancer subtypes. For instance, *Helicobacter pylori*, as a digestive infection bacteria, is reported to promote the initiation and the progression of gastric cancers (Mentis et al., 2019). Although some typical correlations between microorganisms and tumorigenesis are already reported, previous studies are just sporadic studies on single microorganism–cancer subtype pairs but do not explain and validate the specific contribution of microbiome on tumorigenesis.

In March, 2020, a systematic microbiome analyses of blood and cancer-associated tissues in cancer patients/controls reflect the characteristic distribution of the microbiome among different cancer subtypes and their potential contributions to the tumorigenesis procedures (Poore et al., 2020). For the first time, such research has identified some typical signatures of multiple cancer subtypes and tried to identify specific biomarkers with diagnostic or prediction potentials on cancer, confirming that different cancer subtypes have different microbiome profiling patterns. Some optimal biomarkers (microorganisms) from either tumor or blood can be applied for the early diagnosis of certain cancer subtypes. In this study, on the basis of the initial microbiome analyses results, we have further performed two levels of interpretable analyses. On the one hand, we have identified some core regulatory microorganisms that contribute to the classification of multiple tumor subtypes and established quantitative predictive models for accurate prediction by using multiple machine learning methods. On the other hand, we have performed and compared the optimal features (microorganisms) and rules identified from two microbiome profiles [i.e., processed using the Kraken (Wood et al., 2019) and the SHOGUN (Hillmann et al., 2020)] by considering the original study that applied two major sequencing and analysis workflows. Overall, our study has identified new biomarkers and their interpretable classification rules for cancer microbiome discrimination by relying on the systematic analysis of microbiome profiling data and compared the Kraken and the SHOGUN methods for robust cancer microbiome analyses, promoting the development of tumor etiology at microbiome level.

## Materials and Methods

### Data

We downloaded the processed microbiome data of TCGA patients with cancer from ftp://ftp.microbio.me/pub/cancer_microbiome_analysis/(Poore et al., 2020). The data were processed using two different methods, i.e., the Kraken (Wood and Salzberg, 2014) and the SHOGUN (Hillmann et al., 2018). Therefore, two datasets were generated. They all relies on sequence alignment and reference-based taxonomy annotation to identify potential microorganisms from the microbiome data.

The Kraken method can be sequentially divided into three major steps (Wood and Salzberg, 2014):

(1) Mapping k-mers of the query sequence to references of multiple taxonomy.
(2) Identifying all the taxonomies that contain high quality mapped sequences.
(3) Building a weighted classification tree and find the path from root (high level classification category) to leaf (low level classification category) with the highest added score. And the leaf in the classification path with the highest added score is the classification used for the query sequence. The Kraken can also be described as using the k-mers of each sequence to find the lowest common ancestor (LCA) as the final annotation.

As for the SHOGUN method, it can also be divided into three major steps (Hillmann et al., 2018):

(1) Using three methods (Bowtie2, BURST, and UTree) to align the candidate sequence to the genome.
(2) Using weighted last-common ancestor algorithm to annotate each sequence with one taxonomy with confidence generated from all the mapped reads. Further, the BURST aligner can help build a rank-specific relative profiling, finding the most relative profiling of such candidate sequence.
(3) The SHOGUN also applies Bracken algorithm to estimate rank-specific relative abundance using each genome’s uniqueness, profiling hits number and length.

There are two major similarities and three differences between such two computational methods (Wood and Salzberg, 2014; Hillmann et al., 2018).

The similarities include:

(1) The initial step of both methods is mapping to the candidate genomes of multiple microorganisms.
(2) Both methods try to assign one unique taxonomy to each sequence to avoid redundant annotation.

The differences include:

(1) SHOGUN method takes the coverage of target reference and abundance characteristics of the query sequence into consideration to calculate the confidence of annotated taxonomies, while Kraken only control the mapping procedure using aligners’ degree of confidence.
(2) Both methods try to annotate the sequence using one taxonomy, but using different methods: UTree in SHOGUN trying to find “the lowest-common-ancestor scheme” for annotation, while Kraken has its own scoring methods, which search for the root-to-leaf path with the lowest score, taking the entire classification path into consideration not just the final ancestor.
(3) SHOGUN can also evaluate relative abundance of each candidate annotated taxonomies, while Kraken cannot.

As we have presented above, both methods were solid microorganisms identification methods. Considering the differences of such two methods, it is quite reasonable and acceptable for us to identify some different candidate microbiomes for our further classification analyses.

**FIGURE 1:**
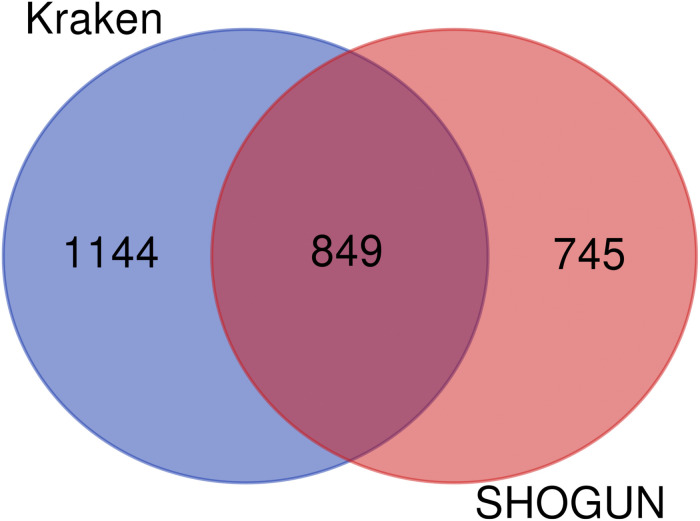
Venn diagram to show the common and different features used in two datasets. Several exclusive features are contained in each dataset.

In the Kraken dataset, 17,625 microbiomes in 1993 samples were obtained from 32 cancer types. In the SHOGUN dataset, 13,517 microbiomes in 1594 samples were obtained from 32 cancer types. We believed that different cancers had different microbiomes, indicating cancer-specific microbiomes. The sample sizes of each cancer type in the Kraken and the SHOGUN datasets are shown in Table 1. Features used to represent samples in each dataset were different. Figure 1 shows the number of common and different features in each dataset. Evidently, each dataset contained several exclusive features. An analysis on these two datasets can give a complete view of different cancer types with microbiome.

**TABLE 1:** Summary of the Kraken and SHOGUN datasets.

Index	Cancer Type	Sample size	
		Kraken dataset	SHOGUN dataset
1	Adrenocortical carcinoma	79	79
2	Bladder urothelial carcinoma	729	729
3	Brain lower grade glioma	731	731
4	Breast invasive carcinoma	1483	1483
5	Cervical squamous cell carcinoma and endocervical adenocarcinoma	451	451
6	Cholangiocarcinoma	45	45
7	Colon adenocarcinoma	1006	417
8	Esophageal carcinoma	340	340
9	Glioblastoma multiforme	489	338
10	Head and Neck squamous cell carcinoma	906	297
11	Kidney chromophobe	191	65
12	Kidney renal clear cell carcinoma	1141	1114
13	Kidney renal papillary cell carcinoma	393	23
14	Liver hepatocellular carcinoma	523	162
15	Lung adenocarcinoma	911	911
16	Lung squamous cell carcinoma	638	534
17	Lymphoid neoplasm diffuse large b-cell lymphoma	61	61
18	Mesothelioma	87	87
19	Ovarian serous cystadenocarcinoma	1031	1031
20	Pancreatic adenocarcinoma	183	183
21	Pheochromocytoma and Paraganglioma	186	186
22	Prostate adenocarcinoma	829	829
23	Rectum adenocarcinoma	372	372
24	Sarcoma	347	347
25	Skin cutaneous melanoma	792	667
26	Stomach adenocarcinoma	1079	1079
27	Testicular germ cell tumors	139	139
28	Thymoma	122	122
29	Thyroid carcinoma	880	287
30	Uterine carcinosarcoma	57	57
31	Uterine corpus endometrial carcinoma	1222	169
32	Uveal melanoma	182	182

### Minimum Redundancy Maximum Relevance (mRMR)

The mRMR (Peng et al., 2005) is a powerful and widely used feature selection method. The informative features evaluated by such method should have (i) minimum redundancy among themselves and (ii) maximum relevance with class labels. To this end, the method employed mutual information (MI) to evaluate the relationships between features or class labels. For two variables *x* and *y*, their MI value can be formulated by

(1) $$I\left(x,y\right)=\iint p\left(x,y\right)\log\frac{p\left(x,y\right)}{p\left(x\right)p\left(y\right)}dxdy$$

where *p*(*x*)/*p*(*y*) stands for the marginal probabilistic density of the variable and *p*(*x*,*y*) indicates the joint probabilistic density of two variables. The mRMR aims to evaluate the importance of features in a list, simultaneously satisfying the above two points. Initially, such list is empty. The feature is selected from the rest features one by one, which has maximum relevance with class labels and minimum redundancy to features already in the list. When all features are in the list, the entire procedures stop. For convenience, the obtained feature list was denoted by *F* in this study.

This study used the mRMR program retrieved from http://penglab.janelia.org/proj/mRMR/. Default parameters were adopted.

### Incremental Feature Selection (IFS)

Although the mRMR method can sort the features with the decreasing order of their importance, it is still difficult to determine which features are essential. This study employed the IFS (Liu and Setiono, 1998) method, which can be used to determine the best number of essential features for a given classification algorithm. At first, IFS produced a series of feature subsets from the above-constructed feature list *F*. For example, the first feature subset consisted of one top-ranked feature, and the second feature subset consisted of two top-ranked features, and so forth. Then, for each feature subset, the IFS trained a classifier on the training samples with features in such set. And this classifier was evaluated by 10-fold cross-validation (Kohavi, 1995). Finally, the IFS determined the optimum feature subset, on which the classification model provided the best performance evaluated by Matthew correlation coefficient (MCC) (Matthews, 1975).

### Synthetic Minority Oversampling Technique (SMOTE)

As listed in Table 1, two microbiome datasets had different numbers of samples in different cancer types. For the Kraken dataset, the largest cancer type had about 33 times samples as many as the smallest type, whereas for the SHOGUN, this number was about 64.5. It is indicated that these two datasets were imbalanced. To reduce the influence of the imbalance, SMOTE (Chawla et al., 2002) was adopted when evaluating the performance of each classification model. This method produces new samples for the minor sample class, thereby ensuring that the number of samples in the minor class was equivalent to that of samples in the major class after an iterative procedure. In detail, it randomly selects a sample, say *x*, in the minor class and finds out some nearest samples to it in the same class. Then, randomly pick up a nearest sample, say *y*, among above nearest samples. A new sample is generated by a linear combination of *x* and *y*. Because the new generated sample has strong associations with *x* and *y*, it has a high probability to be in the same class of *x* and *y*. Thus, it is also assigned such class label. In this study, the SMOTE was employed to enlarge each cancer type except the largest one. Finally, each type has equal number of samples. The “SMOTE” tool available in Weka (Witten and Frank, 2005) was applied in this work.

### Classification Algorithm

Four classification models were used in the microbiome feature learning and rule extraction.

#### Random Forest (RF)

The RF (Breiman, 2001; Wei et al., 2017; Zhao et al., 2018; Baranwal et al., 2019; Jia et al., 2020; Liang et al., 2020) is a tree-based assembly model that predicts the class label of a new sample on the basis of the consensus results of the average predictions from multiple decision trees (DTs). In the present study, we used the RF implemented in the Scikit-learn package.

#### Support Vector Machine (SVM)

The SVM (Cortes and Vapnik, 1995; Sun et al., 2015; Chen et al., 2017; Sang et al., 2020; Zhou et al., 2020a, b) can transform the data point from a low-dimensional data space to a high-dimensional data space. The SVM divides the data samples of each label in the principle of data interval maximization in a high-dimensional space and predicts the class label of a new sample depending on the interval to which this new sample belongs. The SMO algorithm in the Weka software is used to build the SVM model.

#### k-Nearest Neighbor (kNN)

The kNN (Cover and Hart, 1967) first calculates the distance between the test and the training samples and ranks the training samples by using their distance from the test sample. The kNN then selects the *k* high-ranked training samples (i.e., nearest neighbors), estimates the label distribution of such *k* samples, and predicts the label of the test sample as the class label with the highest frequency of the label distribution. The IBk algorithm in the Weka software is used to build the kNN model.

#### DT

The DT (Safavian and Landgrebe, 1991) aims to build the human understanding classification and the regression models by using interpretative rules in a white box model, e.g., using the IF–TEHN format to describe individual features roles and weights in the classification and the regression models. The CART algorithm with the Gini index in the Scikit-learn package was used to build the DT model.

### Performance Evaluation

The MCC (Matthews, 1975), which can evaluate the performance of the classification model, has values from −1 to +1 and achieves +1 when one classification model has the best performance. In this work, the multiclass version of the MCC (Gorodkin, 2004) was applied because the analyzed microbiome data were organized as multiple categories and can be calculated as:

(2) $$MCC=\frac{cov\left(X,Y\right)}{\sqrt{cov\left(X,X\right)cov\left(Y,Y\right)}},$$

where the binary matrix *X* indicates the predicted class of each sample, the binary matrix *Y* represents the true classes of all samples, and *cov*(*X*,*Y*) represents the covariance of two matrices.

## Results

**FIGURE 2:**
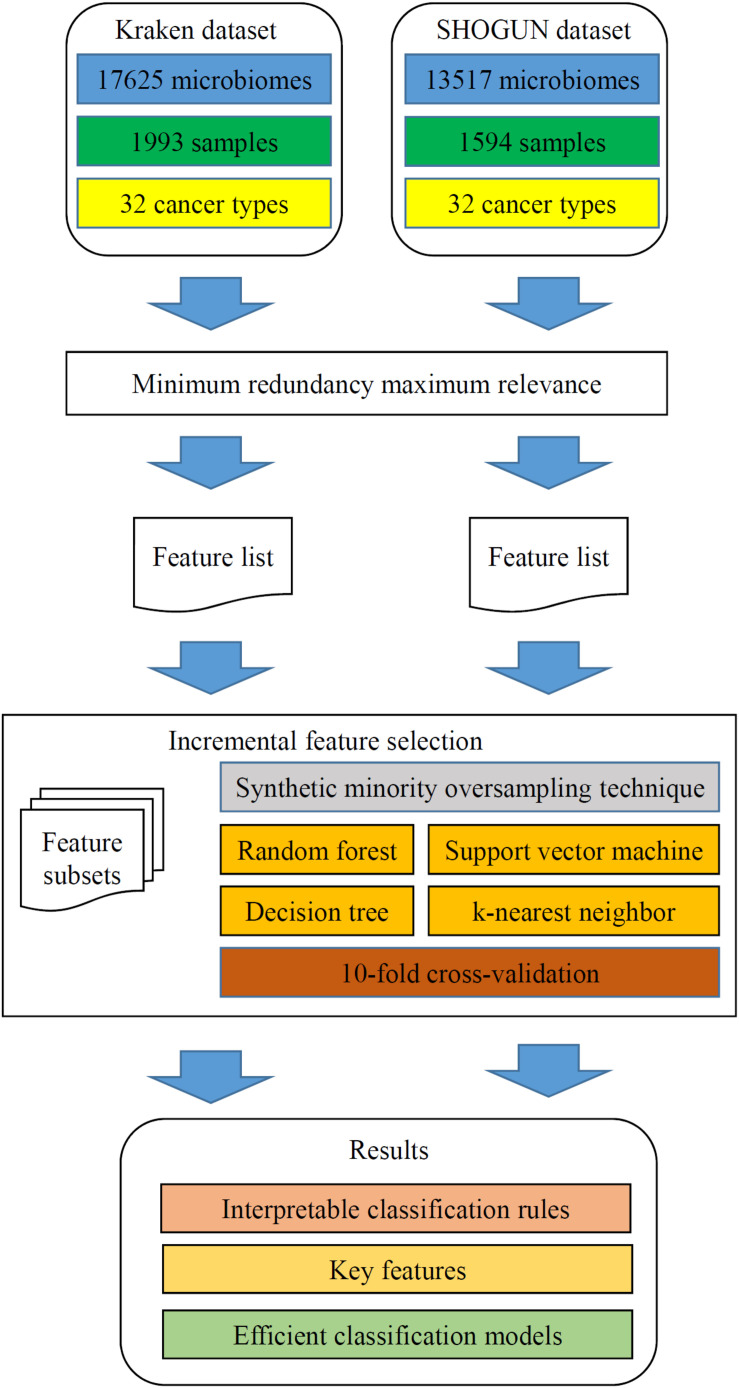
Flow chart to show the detailed analysis procedures. The two datasets are analyzed by minimum redundancy maximum relevance, resulting in a feature list for each dataset. The incremental feature selection, which incorporates synthetic minority oversampling technique, four classification algorithms and 10-fold cross-validation, is applied to each feature list. The results include interpretable classification rules, key features and efficient classification models.

In this study, we analyzed two microbiome datasets using several computational methods. The entire procedures are illustrated in Figure 2.

### mRMR Results

The Kraken and the SHOGUN datasets were all analyzed by the mRMR method. As a result, two feature lists were produced, which are available in Supplementary Tables S1, S2, respectively.

### IFS Results

The feature lists were obtained by applying mRMR method to the Kraken and the SHOGUN datasets, which were fed into the IFS with four classification algorithms.

**FIGURE 3:**
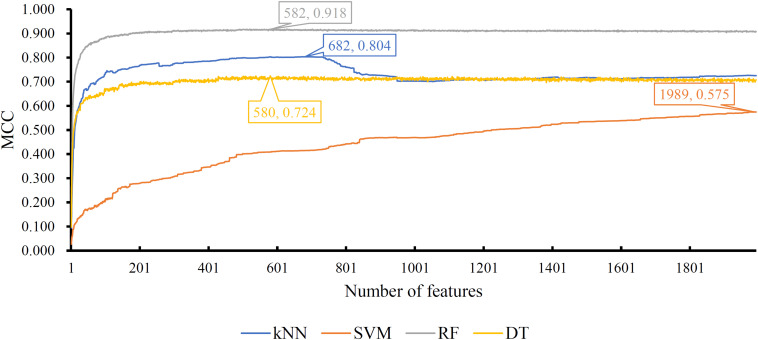
IFS curves yielded by models with different classification algorithms on the Kraken dataset. The random forest with the top 582 features produces the highest MCC of 0.918.

**FIGURE 4:**
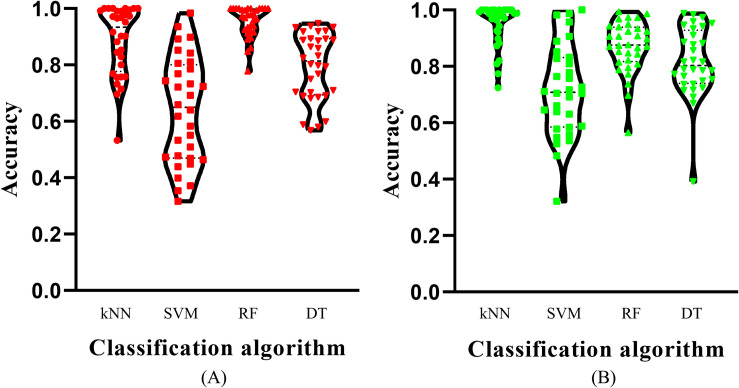
Violin plot to show the accuracies on cancer types which are produced by the best model with different classification algorithms on two datasets. **(A)** Kraken dataset, the RF model yields the most high accuracies; **(B)** SHOGUN dataset, the kNN model produces the most high accuracies.

Of the feature list on the Kraken dataset, we constructed several models with one classification algorithm and some top features in the list. Each model was assessed by 10-fold cross-validation. Obtained measurements, including accuracy on each cancer type, overall accuracy (ACC) and MCC, are provided in Supplementary Table S3. For an easy observation, an IFS curve was plotted for each classification algorithm with MCC as the *Y*-axis and number of features as the *X*-axis, which is illustrated in Figure 3. When RF was selected as the classification algorithm, the highest MCC was 0.918. It was obtained based on the top 582 features. The highest MCCs for the other three algorithms were 0.804, 0.724, and 0.575, respectively, which were based on top 682, 580, and 1989 features. These highest MCCs and corresponding ACCs were collected in Table 2. Evidently, the RF with top 582 features was the best model among all tested models. In addition, the accuracies on 32 cancer types yielded by above best models with different classification algorithms are shown in Figure 4A. Clearly, The RF model yielded higher accuracies on cancer types than those obtained by other models. We called the 582 features used in such RF model as the global optimum features on the Kraken dataset.

**TABLE 2:** Summary of the performance of the best model with different classification algorithms on two datasets.

Classification algorithm	Kraken dataset			SHOGUN dataset		
	Number of features	ACC	MCC	Number of features	ACC	MCC
Random forest	582	0.921	0.918	146	0.884	0.878
Support vector machine	1989	0.588	0.575	1592	0.633	0.616
k-nearest neighbor	682	0.812	0.804	277	0.895	0.889
Decision tree	580	0.736	0.724	1481	0.824	0.814

**FIGURE 5:**
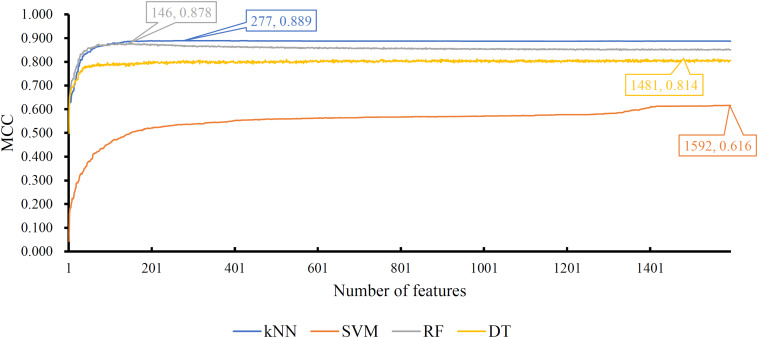
IFS curves yielded by models with different classification algorithms on the SHOGUN dataset. The k-nearest neighbor with top 227 features generates the highest MCC of 0.889.

For the feature list on the SHOGUN dataset, same procedures were done. The performance of all tested models is listed in Supplementary Table S4. Also, four IFS curves were plotted, as shown in Figure 5. It can be observed that the highest MCCs for different classification algorithms were 0.889, 0.878, 0.814, and 0.616, respectively, which were based on top 277, 146, 1481, and 1592 features, respectively. Above MCCs and corresponding ACCs are listed in Table 2. Among these best models with different classification algorithms, the kNN with top 277 features was the best. To further confirm this fact, the accuracies on 32 cancer types yielded by the best models using different classification algorithms are shown in Figure 4B. Evidently, the accuracies produced by the kNN model were in higher levels than those produced by other models. Accordingly, these 277 features were called global optimum features on the SHOGUN dataset.

**FIGURE 6:**
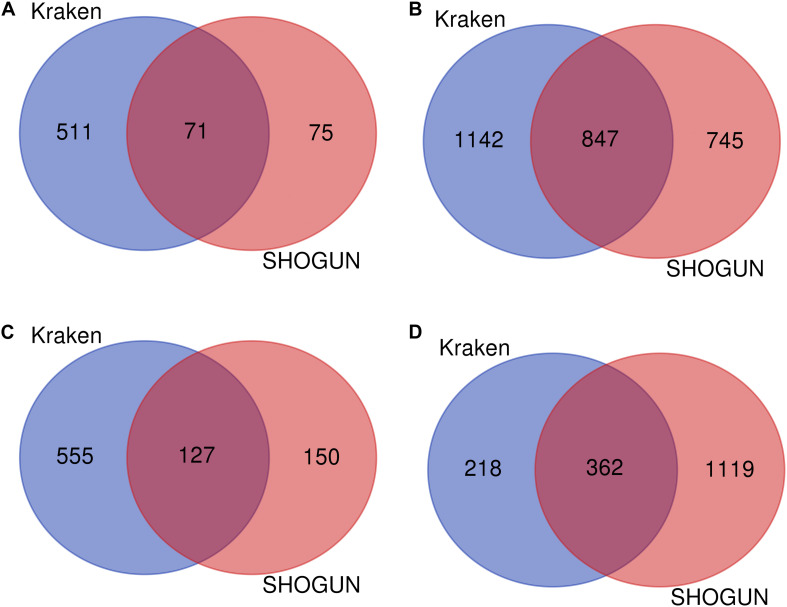
Venn diagram to show the common and difference of optimum feature subsets by applying a given classification algorithm to each of two datasets. **(A)** Random forest; **(B)** Support vector machine; **(C)** k-nearest neighbor; **(D)** Decision tree.

**FIGURE 7:**
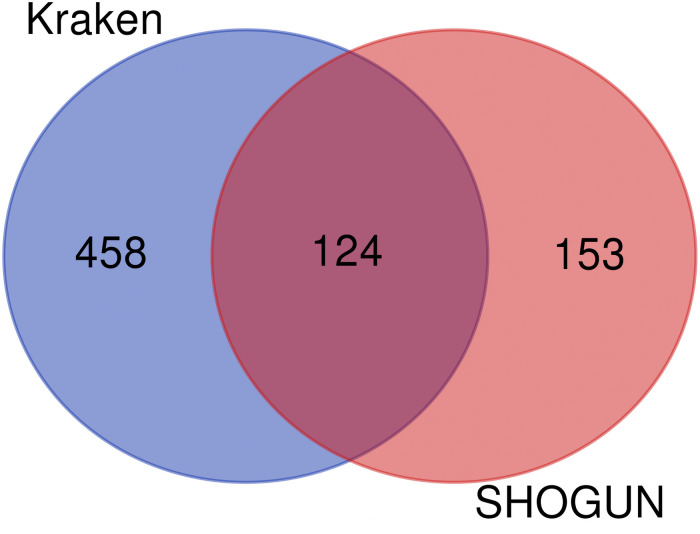
Venn diagram to show the common and difference of the global optimum feature subsets on two datasets.

Given a classification algorithm, IFS method can detect its optimum features on each dataset. In detail, for RF, 582 and 146 optimum features were extracted from Kraken and SHOGUN datasets, respectively. A Venn diagram was plotted to show the common and difference of these two feature sets (Figure 6A), from which we can see that several exclusive features were extracted from each dataset. Similar situations occurred for other three classification algorithms (see Figures 6B–D). Besides, we also analyzed the common and difference of the global optimum features on two datasets (Figure 7). Also, several exclusive features were obtained for each dataset. Above results indicated that the two datasets can provide different information on cancer type at microbiome level. Analyzing them together can give a more complete view on such problem.

### Classification Rules

As mentioned in section “IFS Results,” DT can provide the highest MCC on the Kraken dataset when top 580 features were used. Thus, we applied DT on the Kraken dataset, in which samples were represented by these features. As a result, 3579 rules were obtained, which are provided in Supplementary Table S5. Each cancer type was related to some rules. Rules (310) on cancer type “Breast Invasive Carcinoma” were most, while those (10) on “Uterine Carcinosarcoma” were least. The number of rules related to each cancer type is listed in column 3 of Table 3.

**TABLE 3:** Number of rules for each cancer type on two datasets.

Index	Cancer Type	Number of rules	
		Kraken dataset	SHOGUN dataset
1	Adrenocortical carcinoma	24	8
2	Bladder urothelial carcinoma	235	168
3	Brain lower grade glioma	128	53
4	Breast Invasive carcinoma	310	173
5	Cervical squamous cell carcinoma and endocervical adenocarcinoma	91	89
6	Cholangiocarcinoma	12	13
7	Colon adenocarcinoma	217	112
8	Esophageal carcinoma	55	33
9	Glioblastoma multiforme	31	18
10	Head and Neck squamous cell carcinoma	228	85
11	Kidney chromophobe	40	16
12	Kidney renal clear cell carcinoma	164	104
13	Kidney renal papillary cell carcinoma	132	22
14	Liver hepatocellular carcinoma	122	60
15	Lung adenocarcinoma	232	150
16	Lung squamous cell carcinoma	143	135
17	Lymphoid neoplasm diffuse large b-cell lymphoma	15	18
18	Mesothelioma	19	32
19	Ovarian serous cystadenocarcinoma	59	35
20	Pancreatic adenocarcinoma	65	59
21	Pheochromocytoma and Paraganglioma	26	29
22	Prostate adenocarcinoma	203	137
23	Rectum adenocarcinoma	89	86
24	Sarcoma	56	68
25	Skin cutaneous melanoma	210	94
26	Stomach adenocarcinoma	129	69
27	Testicular germ cell tumors	38	23
28	Thymoma	33	14
29	Thyroid carcinoma	199	101
30	Uterine carcinosarcoma	10	10
31	Uterine corpus endometrial carcinoma	228	11
32	Uveal melanoma	36	5
Total	–	3579	2030

Of the SHOGUN dataset, DT with top 1481 features was best. Accordingly, we applied DT on this dataset, where samples were represented by these 1481 features. As a result, 2030 rules were accessed. These rules are available in Supplementary Table S6. In such rule group, rules (173) on cancer type “Breast Invasive Carcinoma” were still most, while rules (5) on cancer type “Uveal Melanoma” were least. The number of rules related to each cancer type is listed in column 4 of Table 3.

Detailed investigation on above rules can improve our understanding on different cancer types at microbiome level. Some rules were analyzed in section “Discussion.”

## Discussion

Here, we have identified the essential microorganisms for the distinction of different tumor subtypes on the basis of the optimal features produced from two microbiome computational methods (i.e., the Kraken and the SHOGUN). According to recent publications, all predicted microorganisms with distinctive capacity are validated and functionally correlated with one or multiple tumor subtypes. Apart from such qualitative analysis to identify potential tumor subtyping signatures, we have further built up a group of quantitative rules for detailed tumor classification, and these rules are also supported by related literature and datasets in the public domain. The detailed analyses on the optimal features (microorganisms) and their rules can be seen below.

### Key Features Identified From the Microbiome Data Produced by the Kraken Method

The first microorganism in our prediction list on the Kraken data is *Robiginitomaculum* from the specific genus named ***Hyphomonadaceae*.** According to recent publications, a research preprint from the bioRxiv has confirmed that such microorganism may share some similar sequences with the Sonic hedgehog factors in multiple animals and contribute to the internal regulation of related signaling pathways due to such sequence similarities (Jägers and Roelink, 2019). Sonic hedgehog factors are key regulators for the hedgehog signaling pathway, which has been widely reported to contribute to multiple cancer subtypes, including **basal cell carcinomas** (Daya-Grosjean and Couvé-Privat, 2005), **prostate cancer** (Datta and Datta, 2006), and **pancreatic cancer** (Nakashima et al., 2006) with different expression profiling. Therefore, such bacteria can contribute to the detailed classification of multiple cancer subtypes, thereby validating the efficacy and the accuracy of our prediction.

The next predicted microorganism, i.e., ***Mycoplasma***, is a specific kind of microorganism without cell wall around the cell membrane. *Mycoplasma*, as a unique kind of microorganism, is reported to be correlated with multiple cancer subtypes with infections detected either in blood [like cervical cancer (Zhu et al., 2007)] or tumor *in situ* [like **prostate** (Barykova et al., 2011), **gastric** (Yang et al., 2010), and **ovarian** (Chan et al., 1996) **cancers**]. Furthermore, the detailed mechanisms for *Mycoplasma* contributing to tumorigenesis are reported. The microorganism can directly cause pathological chromosomal loss and translocations in multiple cell subtypes (Chan et al., 1996).

The third tumor-associated pathogen, ***Lachnoclostridium*,** is predicted to be functionally correlated with tumorigenesis and may further participate in the detailed tumor classification. According to recent publications, as one of the most famous member of the gut microbiome, these bacteria are functionally correlated with colorectal adenoma and cancer (Liang et al., 2020). Another independent study further validates that such microorganism may even contribute to the non-invasive detection of **colorectal cancer** (Mangifesta et al., 2018), implying that such bacteria can act as an effective classification parameter to identify colorectal cancers from other cancer subtypes.

***Achromobacter*** and ***Acidithiobacillus*** are the next two predicted microorganisms identified on the Kraken data and predicted to be essential classification parameters by our newly presented computational methods. As a pathogen for respiratory tract infection, *Achromobacter* is reported to be correlated with multiple cancer subtypes related to the respiratory tract (Barragán et al., 2018; Nolley et al., 2019), confirming its potential contribution on cancer subtyping. Similarly, *Acidithiobacillus* infects lung cells and contributes to the initiation of **lung cancer** (Ramírez-Aldaba et al., 2017) but not to other cancer subtypes.

### Rules Identified From the Microbiome Data Produced by the Kraken Method

Apart from such qualitative analyses on the mapping and annotation results following the Kraken data, we have identified some quantitative rules for the identification of certain cancer subtypes.

Among such rules, a specific rule contributing to the identification of breast cancer is established with multiple quantitative parameters, including *Succinimonas* and *Campylobacter*. These two microorganisms are chosen as typical parameters for detailed discussion. The microorganism named *Succinimonas* is functionally correlated with the metabolism of breast lactation in cows and human beings (Elolimy et al., 2018). Considering that the breast lactation metabolism is correlated with breast cancer tumorigenesis (Kim and Wysolmerski, 2016; Wani et al., 2017), this microorganism is regarded as a potential quantitative parameter for breast cancer. Many studies have identified the infection of *Campylobacter* in breast cancer (Korneev et al., 2018; Parida and Sharma, 2019), further validating the efficacy and the accuracy of our prediction.

Apart from breast cancer, the urothelial bladder carcinoma is identified using multiple rules. Among them, in a typical rule, *Acidibacillus* and *Nitrospira* are two typical microorganisms that may contribute to the tumorigenesis of such cancer subtype and may participate in the distinction from other cancer subtypes. According to recent publications, these two microorganisms are identified in biological samples from patients with urothelial bladder carcinoma, indicating that both microorganisms are enriched in urothelial bladder carcinoma-associated tissues (Oliveira, 2014; Weng et al., 2016).

Some specific quantitative parameters are screened for sarcoma. *Collinsella* and *Hepacivirus* are identified to contribute to the progression of such disease. In 2019, *Collinsella* is reported as one of the most important gut microbiota that contribute to the initiation and the progression of sarcoma, and these findings correspond with our prediction rules (Vivarelli et al., 2019). As for *Hepacivirus*, according to recent publications, Kaposi’s sarcoma is functionally correlated with the hepatitis C virus, validating our prediction on the upregulated level of *Hepacivirus* in such tumor subgroup (Ray et al., 1995; Wu et al., 2018).

### Key Features Identified From the Microbiome Data Produced by the SHOGUN Method

A similar analysis is performed on the SHOGUN data, and the first microorganism in our prediction result is ***Caballeronia***, which is widely shown to be functionally correlated with the biosynthesis of D-tagatose (Li et al., 2019). The intake and the metabolism of D-tagatose in human beings are reported to be functionally correlated with the specific cell cycle arrest in hepatocytes (Yamaguchi et al., 2008), which contribute to the initiation and the progression of **hepatocellular carcinoma**. Therefore, such microorganism may be applied to distinguish hepatocellular carcinoma from other cancer subtypes.

The next predicted microorganism can be classified into the ***Gammaproteobacteria*** (order). As for its distinctive contribution on different cancer subtypes with specific distribution patterns in human beings, such microorganism has been identified in the pathogenic tissues of two specific digestive system-associated cancer subtypes, i.e., **colorectal** (Peters et al., 2016) and **pancreatic** (Choy et al., 2018) **cancers**.

Another predicted microorganism named as ***Chlamydia*** is similar with *Mycoplasma* as we have analyzed above and a typical subtype of prokaryotic organisms with severe pathogenic capacity. According to recent publications, such organisms are identified in multiple cancer subtypes, including **cervical cancer** with reproductive tract *Chlamydia* infection (Koskela et al., 2000; Smith et al., 2004) and **lung cancer** with respiratory tract *Chlamydia* infection (Laurila et al., 1997; Littman et al., 2005). Such microorganism can infect exposed mucosal tissues and induce tumorigenesis at the regional infection sites, implying its potential capacity on distinguishing different tumor subtypes via their relationship with mucosal tissues.

Moreover, the predicted microorganism named as *Bradyrhizobium* has quite few reports on its differential correlations with different cancer subtypes (only reported to be detected in the serum samples from cancer patients) (Nordlund et al., 2005). The predicted microorganism named as ***Kurthia*** participates in the malignant tumorigenesis, and its distribution may be functionally correlated with the **colorectal cancer** tumorigenesis. The abundance profiling of such microorganism in gut may contribute to the diagnosis and the prognosis prediction on colorectal cancer at least in a mouse model (Yu et al., 2018).

### Rules Identified From the Microbiome Data Produced by the SHOGUN Method

Apart from such microorganisms predicted as qualitative parameters for cancer subtyping on the SHOGUN data, we have established some effective prediction rules for the accurate prediction of certain cancer subtypes on the basis of the detailed microorganism abundance. The optimal rules and the associated features are discussed below.

Specific rules for ovarian cancers are established. The high abundances of *Oribacterium* and *Selenomonas* are predicted to be correlated with the initiation and the progression of ovarian cancers. For *Oribacterium*, recent publications have confirmed that such microorganism is functionally correlated with the hemorrhagic ovarian cyst syndrome in the pathological ovary (Thackray, 2019). Considering that the hemorrhagic ovarian cyst syndrome is one of the typical precancerous lesions of ovarian cancer, speculating the tight correlations between *Oribacterium* and ovarian cancers is quite reasonable (Brown and Frumovitz, 2014). *Selenomonas* is confirmed to be correlated with multiple cancer subtypes, including oral and ovarian (Al-Hebshi et al., 2019) cancers, and these findings correspond with our rules.

Typical rules contributing to the identification of kidney renal clear cell carcinoma are also identified. Among them, two typical parameters are named as *Terasakiispira* and *Candidatus. Thiodiazotropha* contributes to the identification of such cancer subtype with publication support. *Terasakiispira* is detected in the pathological urinary system, including the malignant transformed urinary system (Schultz et al., 2020), and contributes to abnormal genomic alterations in human beings (Angiuoli et al., 2008).

As the final examples, specific rules about the uterine carcinosarcoma involve multiple parameters, including *Natronococcus* and *Terasakiispira*. Both microorganisms are functionally correlated with tumorigenesis (Angiuoli et al., 2008; Hamidi et al., 2019). Although the lack of direct connections between such two microorganisms with the uterine carcinosarcoma, their upregulation at least confirms the malignant transformation in candidate tissues, validating our prediction.

### Comparison of Features and Rules Identified Between the Kraken and the SHOGUN Methods

The results obtained from two kinds of data are compared. Among 1993 Kraken- and 1594 SHOGUN-based predicted microorganisms, 907 specific species are identified to be shared in both methods, implying the reproducibility and the comparability of the two analytic microbiome methods and validating the efficacy and the accuracy of our new prediction methods. For top predicted microorganisms, the detailed species name may vary, but multiple genera are identified to be shared in the 20 top-ranked microorganisms, like *Mycoplasmataceae*, *Enterococcaceae*, and *Rhodobacteraceae*, which indeed have solid publication support to be correlated with the tumorigenesis of some cancer subtypes.

## Conclusion

Overall, the optimal features and rules in our prediction lists have been validated by recent publications, and they are robust and efficient for different analytic microbiome methods (i.e., Kraken and SHOGUN). Our study has identified a group of novel potential biomarkers/rules for the subgrouping of different cancer subtypes on the microbiome level and provided an effective computational tool to identify the potential associations between microbiome and tumorigenesis, thereby exploring the complicated microenvironment components associated with tumorigenesis.

## Data Availability Statement

Publicly available datasets were analyzed in this study. This data can be found here: ftp://ftp.microbio.me/pub/cancer_microbiome_analysis/.

## Author Contributions

TH and Y-DC designed the study. LC, ZL, TZ, and Y-HZ performed the experiments. LC, ZL, DL, and HL analyzed the results. LC and ZL wrote the manuscript. All authors contributed to the research and reviewed the manuscript.

## Conflict of Interest

The authors declare that the research was conducted in the absence of any commercial or financial relationships that could be construed as a potential conflict of interest.

## Supplementary Material

Supplementary Table S1 Rank of relevant features from mRMR on the Kraken dataset.

Supplementary Table S2 Rank of relevant features from mRMR on the SHOGUN dataset.

Supplementary Table S3 Performance of IFS with different classification models under different numbers of features on the Kraken dataset.

Supplementary Table S4 Performance of IFS with different classification models under different numbers of features on the SHOGUN dataset.

Supplementary Table S5 Rules generated by decision tree on the Kraken dataset.

Supplementary Table S6 Rules generated by decision tree on the SHOGUN dataset.
